# Using Internet Search Trends and Historical Trading Data for Predicting Stock Markets by the Least Squares Support Vector Regression Model

**DOI:** 10.1155/2018/6305246

**Published:** 2018-07-24

**Authors:** Ping-Feng Pai, Ling-Chuang Hong, Kuo-Ping Lin

**Affiliations:** ^1^Department of Information Management, National Chi Nan University, 1 University Rd., Puli, Nantou 54561, Taiwan; ^2^Department of Information Management, Lunghwa University of Science and Technology, No. 300 Sec. 1, Wanshou Rd., Guishan District, Taoyuan 33306, Taiwan; ^3^Institute of Innovation and Circular Economy, Asia University, Taichung 41354, Taiwan

## Abstract

Historical trading data, which are inevitably associated with the framework of causality both financially and theoretically, were widely used to predict stock market values. With the popularity of social networking and Internet search tools, information collection ways have been diversified. Instead of only theoretical causality in forecasting, the importance of data relations has raised. Thus, the aim of this study was to investigate performances of forecasting stock markets by data from Google Trends, historical trading data (HTD), and hybrid data. The keywords employed for Google Trends are collected from three different ways including users' definitions (GTU), trending searches of Google Trends (GTTS), and tweets (GTT) correspondingly. The hybrid data include Internet search trends from Google Trends and historical trading data. In addition, the correlation-based feature selection (CFS) technique is used to select independent variables, and one-step ahead policy is adopted by the least squares support vector regression (LSSVR) for predicting stock markets. Numerical experiments indicate that using hybrid data can provide more accurate forecasting results than using single historical trading data or data from Google Trends. Thus, using hybrid data of Internet search trends and historical trading data by LSSVR models is a promising alternative for forecasting stock markets.

## 1. Introduction

With the advances of the Internet and communication in recent years, the increasing amount of data from social networks leads to changes in ways of collecting and analyzing data. Google Trends (http://www.google.com/trends) can be used to search trends of keywords. Hence, the data from Google Trends data started to be applied to many fields such as economy, election, and medication. Compared to structured data, collection data from social networks are another way to depict the issues concerned, and thus, some other interesting and essential insights that are not included in the traditional data collection may be discovered. Ever since the beginning of the stock market, it is hard to predict. However, the stock markets have profound effects on a country. In the past, the forecasting of stock markets has relied heavily on historical trading data. Most forecasting models using historical trading data are based on the causality theoretically. Due to the popular use of the Internet search, people tend to seek data or information from the Internet and express opinions on social networks. Stephens-Davidowitz [1] indicated that when social censoring issues are studied, Internet search behaviors can better reflect the real thinking of people than survey data, and the timing to obtain data is more close to real time [2–6]. However, the importance of historical trading data in forecasting stock market values should not be disregarded. This study attempts to incorporate the data from Google Trends and historical trading data together to predict stock markets. The performance of hybrid data and the unique data type in forecasting stock market closing values were examined in this investigation. Five stock markets, namely, Dow Jones Industrial Average Index (DJIA), Nasdaq Composite Index (IXIC), Russell 2000 Index (RUT), Standard & Poor's 500 Index (S&P 500), and Chicago Board Options Exchange Volatility Index (VIX), and three companies, the Apple corporation (APPL), the Alphabet corporation (GOOGL), and the Microsoft Corporation (MSFT), were forecasted by least squares support vector machines models with different data types. The rest of this article is organized as follows: Section 2 provides the related work.  Section 3 introduces the methods employed in this study.  Section 4 illustrates the proposed stock-forecasting framework and numerical examples.  Section 5 draws conclusions.

## 2. Related Work

Hassan [7] noted that predicting stock markets using complex calculations does not help much. The author proposed a forecasting technique combining the hidden Markov model and fuzzy concept to predict stock markets. The results showed that the presented model outperformed the autoregressive integrated moving average model, the neural network model, and other hidden Markov models. Hadavandi et al. [8] claimed that a successful forecasting technique model for stock markets is a technique that can obtain accurate forecasting results with the smallest amount of input data and the simplest stock market model. This article combined genetic fuzzy systems and neural networks to forecast stock markets for information technology companies and airline companies. For the data-preprocessing stage, the stepwise regression analysis was used to pick factors, and then, through the self-organizing map approach, they were employed to cluster data. The experiment's results showed that the proposed approach can obtain more accurate results than some other forecasting methods. Singh and Borah [9] designed a forecasting model consisting of fuzzy theory and the particle swarm optimization technique to predict stock markets by using historical data from the State Bank of India. The numerical results illustrated that the proposed forecasting model is superior to the grey model, artificial neural networks, and regression models.

Another tendency of forecasting stock markets is putting finance indicators into forecasting models. Laboissiere et al. [10] developed a model including correlation analysis and artificial neural networks to predict stock prices of Brazilian electric companies. In addition to the historical trading data, some indices such as the Ibovespa index, the Electric Power index, and American dollar quote were employed to predict stock prices. The numerical results were promising in terms of forecasting accuracy. Lincy and John [11] presented a multiple fuzzy inference systems model to predict selected stocks prices of the Nasdaq stock exchange. Four indicators, Moving Average Convergence/Divergence, Relative Strength Index, Stochastic Oscillator, and Chaikin Oscillator, were used by the proposed model, and decision rules were generated by using fuzzy set theory and multicriteria decision-making approaches. Simulation results revealed that the presented model is a positive way to analyze stock prices in terms of profit return. de Oliveira et al. [12] used artificial neural networks to forecast Petrobras' PETR4 stock by fundamental and technical factors which may influence stock markets. After the data-preprocessing procedure, essential factors left out were used by artificial neural networks. This study reported that the testing accuracy of stock market directions was more than ninety percent. Göçken et al. [13] applied metaheuristics, which are employed to select essential indicators, and artificial neural networks in stock price prediction. In addition, this study examined the suitable number of hidden neurons in the hidden layer in order to deal with the overfitting or underfitting problems of artificial neural networks. The results indicated that the proposed forecasting model was a dominant way to predict stock markets.

Because the use of social networks is booming, data from social networks offer valuable insights into what people think and want. Thus, these data have become more and more popular for collecting opinions and for forecasting. Stephens-Davidowitz [1] studied the relation between the voting of American presidential election and racially charged language. The author pointed out that the Google search queries were more useful than the survey data when social censoring issues were investigated. The results showed that there was a relation between voting and the search queries of racial animus. Gunn III and Lester [5] employed Google Trends with three terms to analyze the relation between the three terms and monthly suicide rates. They reported that the information from the Internet search is correlated with the number of suicides, and thus, it is a faster way of monitoring possible suicide trends than compiling suicide statistics. Yang et al. [14] analyzed the relation between Internet search trends and suicide death. The conclusions revealed that suicide-related search terms were related to suicide death, and thus, keyword-driven search results of the Internet are the essential knowledge to reduce suicide deaths. Frijters et al. [4] conducted a study about the relationship between macroeconomic conditions and an indicator of problem drinking data from Google searches. The results showed that the macroeconomic conditions are associated with health in some ways, and the real-time data provided by Google searches are crucial information for policy-makers. Smith [15] investigated the volatility in forecasting foreign currency exchange rates by using three Google search keywords and time-series models. The results demonstrated that the information from Google searches is important in forecasting the market for foreign currency. Fondeur and Karamé [16] used the Google search data to enhance the prediction accuracy of youth unemployment in France. The results indicated that Google search data did improve the prediction of unemployment. Li et al. [17] used both statistical data and Google search data to predict the consumer price index by a mixed-data sampling model. Numerical results revealed that the proposed approach was helpful in forecasting the consumer price index by using data from the user-generated content. Takeda and Wakao [18] studied the relation between the Google search intensity, stock trading volume, and stock prices. It was reported that the positive relationship between Google search intensity and trading volume is stronger than that between Google search intensity and stock prices. Araz et al. [2] used Google Flu Trends data to forecast influenza-like illness, and a strong positive relation between Google Flu Trends data and influenza-like illness was revealed. In addition, using Google Flu Trends data as independent variables can result in accurate forecasting results. Some studies have examined the relation between the Internet search and some diseases, such as disease-related genes [19], kidney stones [20, 21], epilepsy [3, 22], allergy [23], and restless legs [24].

Most data on social networks are unstructured. Therefore, to find meaningful information from social networks, text mining has been one of the major tools employed. Mostafa [25] used tweet samples on some famous companies to analyze sentiments of users to forecast the Prosperity index of each company. This investigation concluded that text mining in social networks is a helpful way to capture consumers' view and preferences of products. Ikeda et al. [26] investigated the Japanese tweeters and developed a hybrid text-based and community-based method for the demographic group or prediction of Twitter users. The proposed method can analyze tweeter's hobby, occupation, marital status, age, gender, and area. The authors reported that the proposed hybrid method can increase the precision of the text-based method. He et al. [27] collected social media data from both their own sites and the competitors' sites in the pizza industry. This study indicated that the social media competitive analysis is essential and can help companies to form marketing strategies. Yu and Wang [28] gathered real-time tweets during 2014 World Cup games and employed text mining tools to distinguish positive and negative comments which may reflect moods of the soccer fans during matches. This study showed that opinions of sports fans can be learned from Twitter, and the results were fairly close to the predictions of the disposition theory. Chae [29] used a collection of Twitter hashtags related to the supply chain to gain some insight into supply chain management. The presented model consists of four approaches, descriptive analytics, content analytics, integrating text mining and sentiment analysis, and network analytics. Some interesting and valuable conclusions have been reached from the studies on the professional use of Twitter, organizational use of Twitter, and supply chain research, respectively.

## 3. Methodology

Proposed by Hall [30], the correlation-based feature selection (CFS) is a feature identification technology used for determining features with critical influence on prediction classes. The influence of features is related to the correlation between the feature and the prediction class labels. The correlation function is represented as follows:

(1) $$\begin{array}{c} {\text{DOF}}_{p}=\frac{\text{NV}{\bar{r}}_{qi}}{\sqrt{\text{NV}+\text{NV}\left(\text{NV}-1\right){\bar{r}}_{ii}}}, \end{array}$$

where DOF_*p*_ is the degree of importance of a feature set *p*, NV is the amount of features in the subset *p*, ${\bar{r}}_{qi}$ is the average correlations between the feature *i* in the subset *p* and the class *q*, and ${\bar{r}}_{ii}$ is the average intercorrelation between features. The best-first search algorithm [31] was employed to generate the appropriate feature subset, and the Weka [30, 32] software was utilized to perform CFS in this investigation. The support vector machines [33, 34] model has been one of the most prevalent classification techniques in the past two decades. The support vector machines model was extended to cope with regression problems, and the support vector regression [35–37] has become popular in solving function approximation problems. Both support vector machines and support vector regression have to handle quadratic functions during the problem-solving processes. This is a time-consuming task. This restriction has been overcome by transferring a quadratic programming problem into a linear equation so that it can be solved. The least square support vector regression (LSSVR) [38] model can be represented as follows:

(2) $$\begin{array}{c} \begin{array}{cc} \text{Minimize} & F\left(w,\xi \right)=\frac{1}{2}w^{T}w+\frac{1}{2}ϒ\overset{m}{\underset{i=1}{\sum }}\xi_{i}^{2},\\ \text{subject}\;\;\mathrm{to} & y_{i}=w^{T}\cdot \emptyset \left(x_{i}\right)+p+\xi_{i},\;i=1,\ldots ,\;\;m, \end{array} \end{array}$$

where *w* is the weighted vector or the normal of the hyperplane, *ϒ* is the penalty parameters that manipulate the balance between the minimization of estimation error and smoothness of the estimated function, *ξ*_*i*_ is the error vector of the *i*th sample point, ∅(*x*_*i*_) is the nonlinear function mapping of *x*_*i*_ from the original space into a high dimension feature space, *p* is the bias parameter, and *y*_*i*_ and *x*_*i*_ are input data and output value, respectively.

Due to the difficulty of solving the optimization problem straightly, the Lagrange function is developed and the dual problem can be represented as follows:

(3) $$\begin{array}{c} L\left(w,\;\;p,\;\;\nu ,\;\;\xi \right)=F\left(w,\xi \right)-\overset{m}{\underset{i=1}{\sum }}\nu_{i}\left(W^{T}\cdot \emptyset \left(x_{i}\right)+p+\xi_{i}-y_{i}\right), \end{array}$$

where *ν*_*i*_ are the Lagrange multipliers.

By solving the above functions, the solution of the problem can be achieved when all derivatives are equal to zero based on the Karush–Kuhn–Tucker conditions [39–41]. The optimal conditions are shown as follows:

(4) $$\begin{array}{c} w=\overset{m}{\underset{i=1}{\sum }}\nu_{i}\emptyset \left(x_{i}\right),\\ \overset{m}{\underset{i=1}{\sum }}\nu_{i}=0,\\ \nu_{i}=ϒ\xi_{i},\\ w^{T}\emptyset \left(x_{i}\right)+p+\xi_{i}-y_{i}=0. \end{array}$$

By removing *w* and *ξ*_*i*_ from (4), the following linear equation can be obtained:

(5) $$\begin{array}{c} \left[\begin{array}{cc} 0 & I^{T}\\ I & K+{ϒ}^{-1}I \end{array}\right]\left[\begin{array}{c} p\\ \nu \end{array}\right]=\left[\begin{array}{c} 0\\ y \end{array}\right], \end{array}$$

where *I*=[1,1,…,1]^*T*^.

K is a kernel matrix and determined by

(6) $$\begin{array}{c} K_{i,j}=\emptyset {\left(x_{i}\right)}^{T}\cdot \emptyset {\left(x_{j}\right)}^{T}=K\left(x_{i},x_{j}\right), \end{array}$$

where *K*(*x*_*i*_, *x*_*j*_) indicates the kernel function satisfying the Mercer's condition [42].

In this study, the radial basis function represented by (7) was employed as a kernel function:

(7) $$\begin{array}{c} K\left(x_{i},\;\;x_{j}\right)=\exp\;\;\left(-\frac{{\left\|x_{i}-x_{j}\right\|}^{2}}{2\sigma^{2}}\right), \end{array}$$

where *σ* is the kernel width. By solving (5), *v*_*i*_ and *p* can be obtained, and the LSSVR function is represented as follows:

(8) $$\begin{array}{c} y=\overset{n}{\underset{i=1}{\sum }}v_{i}K\left(x,x_{i}\right)+p. \end{array}$$

## 4. The Proposed Stock Market-Forecasting Framework and Numerical Examples

### 4.1. The Proposed Framework

**Figure 1:**
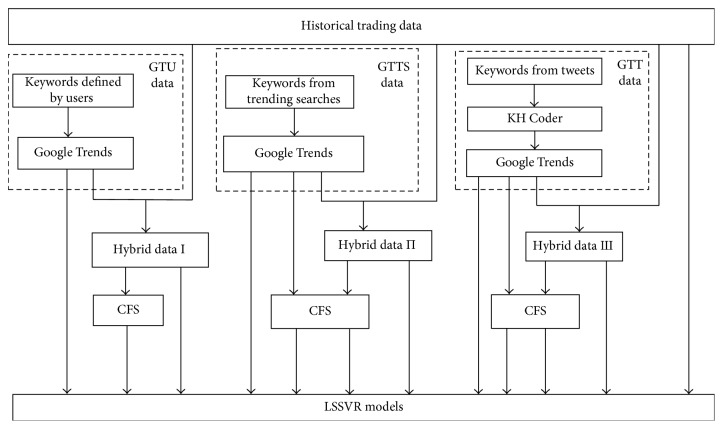
The proposed stock market-forecasting framework.

Figure 1 shows the framework of this study. Three major types of data, namely, data from Google Trends, historical trading data, and hybrid data, were gathered in this study. When using Google Trends data as independent attributes for making a forecast, the determination of related search keywords influences forecasting results a lot. Thus, in this study, keywords of Google Trends were collected in three ways: users' definitions (GTU), trending searches of Google Trends (GTTS), and tweets (GTT), respectively. Firstly, for collecting GTU data, users specified keywords subjectively with some domain knowledge or intuition. Secondly, keywords of Google Trends were gathered by the GTTS approach. Google Trends has a way to calculate keywords' activity levels, namely, trending searches of Google Trends. When a specific term is considered, the results show other related keywords from the highest activity level to the lowest one. Then, the keywords of trending searches are ranked. Users can select keywords in terms of the ranking. The third way of generating keywords for Google Trends is the GTT method which collects texts on Twitter. When keywords for Google Trends obtained from Twitter were employed, the word “clusters tool” provided by KH Coder [43] was employed in this study to select the first 100 terms according to the scores calculated. For three methods of generating keywords for Google Trends, only keywords for Google Trends with scores were used as independent variables to forecast stock markets in this study. Some keywords for Google Trends are without scores due to the low search frequencies. Three hybrid data sets shown in Table 1 were generated by combining the historical data set data set with three data sets of Google Trends. Hybrid data I, hybrid data II, and hybrid data III represent historical data with data of GTU, GTTS, and GTT correspondingly.

Then, the correlation-based feature selection technique was performed for determining essential independent variables to predict stock markets. Since GTU data and historical trading data are with a small number of features, all data sets except the GTU data and historical trading data were processed by the feature selection procedure. Therefore, totally 12 types of independent variables were used in this study to forecast stock markets. One-step ahead policy was employed to predict values of stock markets for all data sets. All 12 types of data were divided into three parts, namely, training data, validation data, and testing data, for LSSVR models to predict five stock markets. The training and validation data were used to select the LSSVR models, and the testing data were utilized to evaluate the forecasting performance of LSSVR models. In addition, genetic algorithms [44] were employed to determine parameters of LSSVR models [45]. In addition, the mean absolute percentage error (MAPE) and mean absolute error (MAE) were used to measure the performance of LSSVR models. The MAPE can be represented as follows:

(9) $$\begin{array}{c} \text{MAPE}\;\;\left(\%\right)=\frac{100}{N}\overset{N}{\underset{t=1}{\sum }}\left|\frac{A_{t}-F_{t}}{A_{t}}\right|, \end{array}$$

(10) $$\begin{array}{c} \text{MAE}=\frac{1}{N}\overset{N}{\underset{t=1}{\sum }}\left|A_{t}-F_{t}\right|, \end{array}$$

where *N* is the number of forecasting periods, *A*_*t*_ is the actual value at period *t*, and *F*_*t*_ is the forecasting value at period *t*.

### 4.2. Numerical Examples

Five daily data sets of stock markets, Dow Jones Industrial Average Index (DJIA), Russell 2000 Index (RUT), Standard & Poor's 500 Index (S&P 500), Volatility Index (VIX), and Nasdaq Composite Index (IXIC), and three companies, the Apple corporation (APPL), the Alphabet corporation (GOOGL), and the Microsoft Corporation (MSFT), obtained from Yahoo Finance (http://finance.yahoo.com) were employed in this study. The data from Google Trends and historical trading data of the current working days were used to predict the stock market values or stock prices of the next working day. Due to the function limitation of Google Trends, the daily search data can be collected within the time horizon of 270 days. Within the limited time horizon of 270 days excluding weekends and national holidays, the data of working days were gathered and one-step ahead policy was employed to predict values of stock markets for all data sets. The time period of the Google Trends data and historical trading data is from June 14, 2016, to March 9, 2017, and data were divided into the training data set (from June 14, 2016, to December 9, 2016), the validation data set (from December 12, 2016, to January 25, 2017), and the testing data set (from January 26, 2017, to March 9, 2017). The training data set, validation data set, and testing data set contain 126, 30, and 30 data, respectively. For the data from Google Trends, three types of data, namely, GTU data, GTTS data, and GTT data, were used in this study. The Google Trends search keywords determined by users, trending searches, and tweets are listed in Tables 2 [Table tab3]–4, respectively. When the GTT data were collected, terms of five stock markets and three corporations, namely, Dow Jones Industrial Average Index, Russell 2000 Index, S&P 500, Volatility Index, Nasdaq Composite Index, APPL, GOOGL, and MSFT, were searched by the Twitter search engine and related tweets were determined. Then, KH Coder [43] was used as a text mining tool to select terms from tweets. The top 100 terms provided by the KH Coder were put into the Google Trends search. Not all keywords selected from KH Coder could be observed from the Google Trends search due to the shortage of search volume. Sequentially, the CFS was performed to select essential keywords of Google Trends determined by trending searches and by tweets. The results are shown in Tables 5 and 6, respectively.

**Figure 2:**
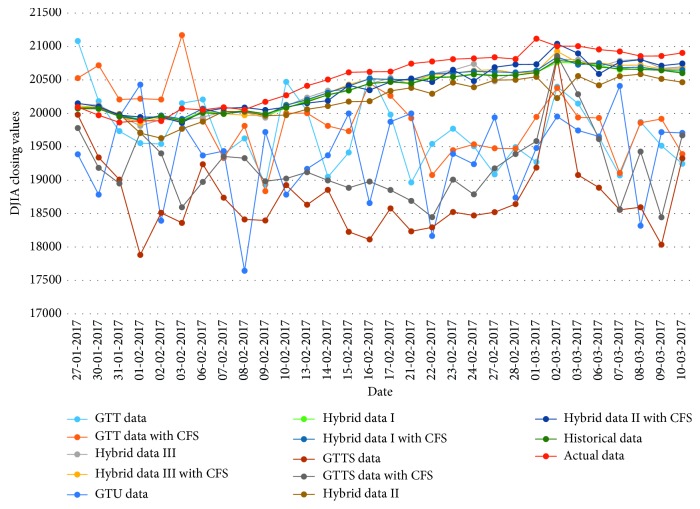
Closing values of the DJIA stock market.

**Figure 3 fig3:**
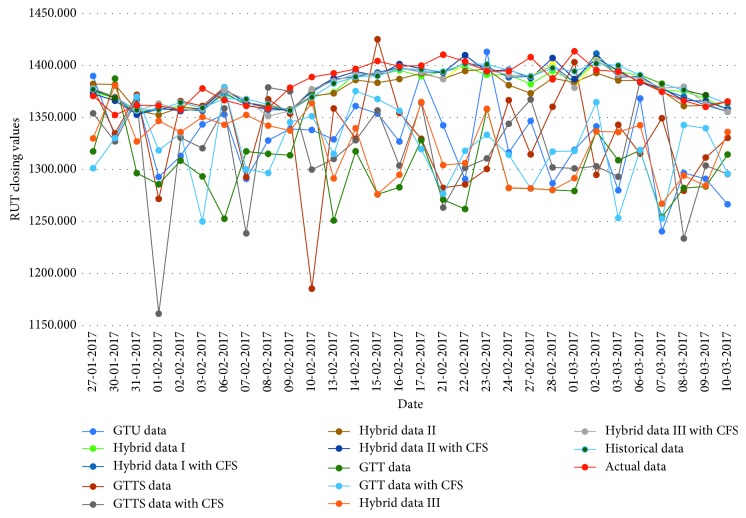
Closing values of the RUT stock market.

**Figure 4 fig4:**
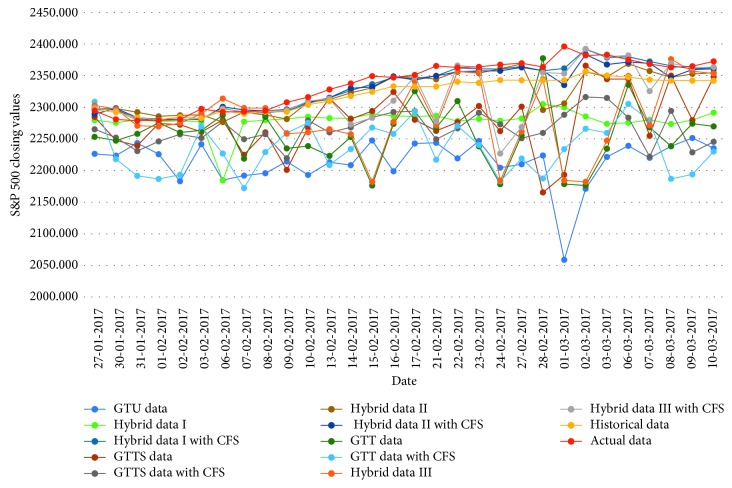
Closing values of the S&P 500 stock market.

**Figure 5 fig5:**
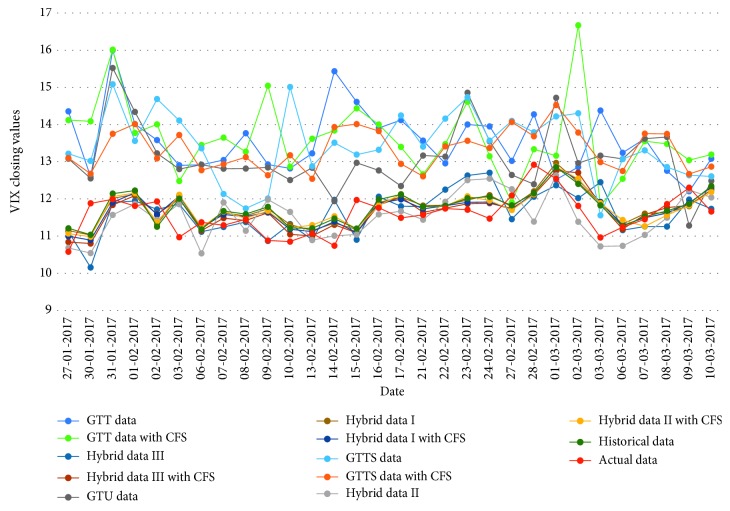
Closing values of the VIX stock market.

**Figure 6 fig6:**
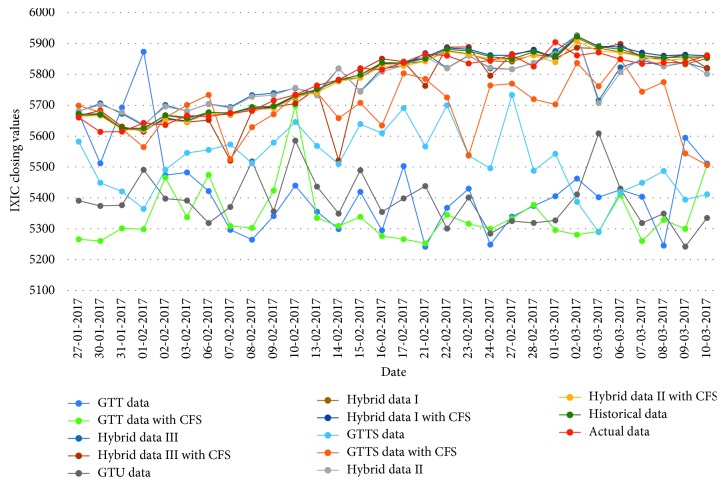
Closing values of the IXIC stock market.

**Figure 7 fig7:**
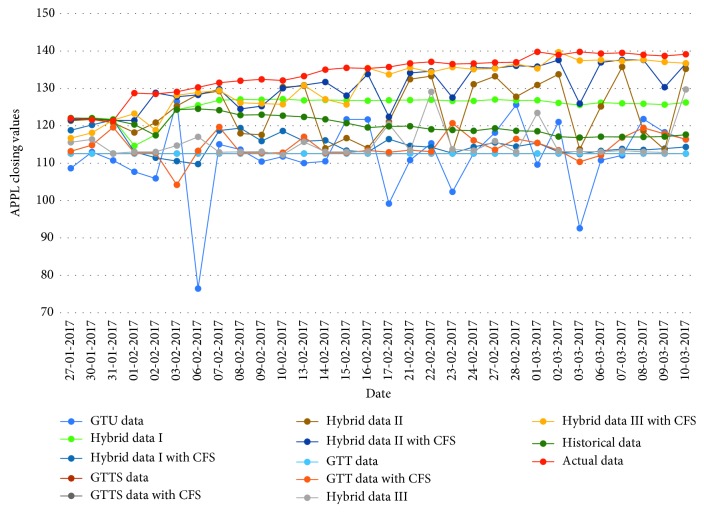
Closing values of the Apple corporation.

**Figure 8 fig8:**
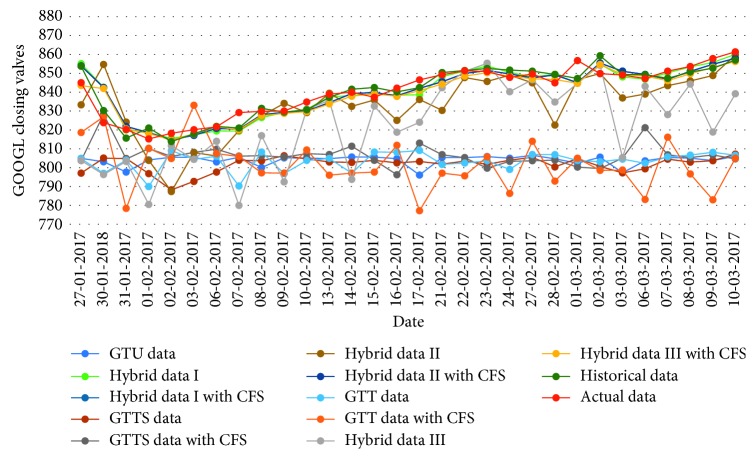
Closing values of the Alphabet corporation.

**Figure 9:**
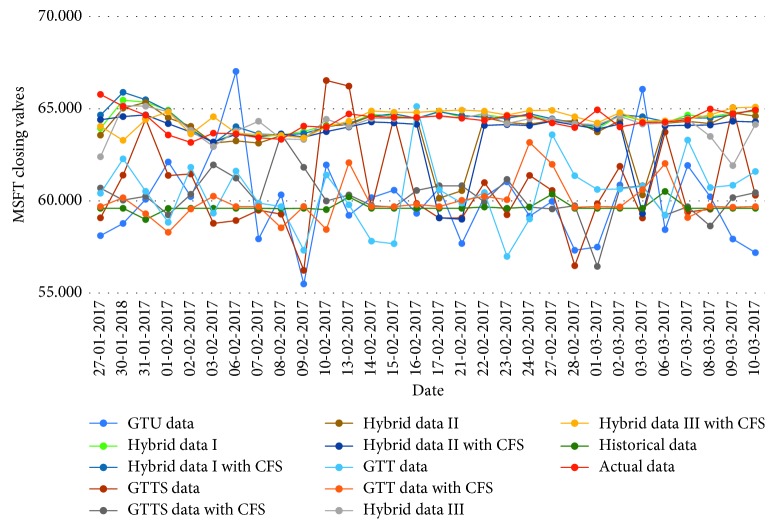
Closing values of the Microsoft Corporation.

Five variables, including opening values, maximum values, minimum values, closing values, and trading volume, were used as condition variables, and closing values of the next day were used as the variables predicted [7–9,12,46]. Three types of hybrid data were used to predict stock markets. Tables 7 [Table tab8]–9 show the selected keywords and historical data attributes of three hybrid data used for five stock markets by using CFS. Tables 10 [Table tab11] [Table tab12] [Table tab13] [Table tab14] [Table tab15] [Table tab16]–17 indicate testing MAPE and MSE values and two LSSVR parameters of different data types for predicting five stock markets and three corporations. The point-to-point comparisons of actual and predicted values by using various data to forecast values of stock markets and corporations are presented in Figures 2 [Fig fig3] [Fig fig4] [Fig fig5] [Fig fig6] [Fig fig7] [Fig fig8]–9. The experiment's results revealed that using hybrid data with LSSVR models does improve forecasting performance on closing values of five stock markets and three corporations.

## 5. Conclusions

Many forecasting models have been proposed for stock market forecasting in the past decades. Due to the rise of social networking and Internet search tools, types of data employed for predicting stock markets became diversified. This study proposed a framework to explore the influence of Internet search trends, historical trading data, and hybrid data on the prediction of stock markets by the least squares support vector regression models. Numerical experiments indicate that using hybrid data can provide satisfied forecasting results. The superior performance and success of the proposed framework are most likely owing to employing the unique advantage of data from the Internet search and historical trading data. Empirically, the Google data may capture a part of the nonlinear data patterns [47], and therefore, the variety of the data has a chance to improve the forecasting performance. The promising results achieved in this study reveal the potential of the proposed framework for forecasting stock markets.

Since keywords of Google Trends significantly affect the forecasting accuracy, Naccarato et al. [48] pointed out the selection of keywords results in different data sets for analysis and thus generates different numerical results. This study provided three ways, namely, users' definitions, trending searches of Google Trends, and tweets, to determine keywords for Google Trends. The three ways can be easily and systematically reproduced for future use. Some other advanced techniques for determining appropriate keywords for Google Trends could be an essential direction for future study. In addition, numerical examples in the developed markets were employed to depict the proposed framework. For emerging markets, owning to the restriction of languages used for Twitter and Google Trends, some hurdles have to be overcome for analyzing the performance of the proposed framework.

## Figures and Tables

**Table 1:** Three hybrid data sets.

Hybrid data types	Components
Hybrid data I	GTU data and HTD
Hybrid data II	GTTS data and HTD
Hybrid data III	GTT data and HTD

**Table 2:** Google Trends search keywords determined by users.

Stock markets/corporations	Keywords
DJIA	dow jones, dow jones average, dow jones industrial
RUT	russell 2000, russell index, russell index 2000
S&P 500	s&p 500, s&p 500 index, standard & poor's 500
VIX	vix, vix index, vix s&p 500
IXIC	nasdaq, nasdaq stock, nasdaq-stock exchange
APPL	aapl, apple stock, aapl stock
GOOGL	alphabet, alphabet stock, google stock
MSFT	msft, microsoft stock, msft stock

**Table 3 tab3:** Google Trends search keywords determined by trending searches.

Stock markets/corporations	Keywords
DJIA	dow jones industrial average, dow jones industrial average today, dow jones industrial average stocks, apple stock, euro to dollar, spdr dow jones industrial average etf, aapl, lowes stock, ftse 100, us dollar to mexican peso exchange rate, tenneco stock

RUT	russell 2000 index, etf russell 2000, russell 2000 stock, russell 2000 index fund, russell 2000 futures, s&p 500, vanguard russell 2000, russell 2000 value, ishares russell 2000, s&p 500 index, russell 2000 stock price, russell 2000 today, russell 2000 growth index, russell 2000 components, s&p 400

S&P 500	s&p 500 index, s&p 500 stock, etf s&p 500, etf, s&p 500 futures, s&p 500 index fund, vanguard s&p 500, vanguard, dow, s&p 500 returns, s&p 500 funds, stocks, s&p 500 stocks, s&p 500 price, stock market s&p 500, stock market, dow jones, what is s&p 500, s&p 500 chart, s&p 500 index funds, index funds, nasdaq, spdr s&p 500, spdr, s&p 500 companies

VIX	vix,volatility, vix futures, volatility index, vix index, vix options, vix volatility, cboe, vix stock, s&p 500, vix trading, cboe vix, s&p 500 vix, vix chart, vix etf, vxx, cboe volatility index, india vix, vix future, spx, volatility s&p 500

IXIC	nasdaq composite, stock, nasdaq stock, stocks nasdaq, stocks, nasdaq market, nasdaq stock market, stock market, nasdaq index, dow, nasdaq futures, dow jones, what is nasdaq, nasdaq omx, oil nasdaq, nasdaq 100, nasdaq today, nasdaq etf, nasdaq stock price, nyse, s&p 500, oil price, nasdaq oil price, nasdaq chart, penny stocks nasdaq, nasdaq stock exchange

APPL	stock aapl, aapl stock price, aapl stock price today, aapl stocks, dow, tsla stock, aapl options, aapl futures, aapl dividend, qqq, aapl nbc, amd stock, aapl chart, aapl ticker, spy stock, aapl stock news, aapl dividend date, amazon stock, aapl options chain, aapl option chain, aapl stock quote, aapl outstanding shares, twlo stock, aa stock, nyse aapl, aapl

GOOGL	stock googl, googl stock price, amzn stock, amzn, amazon stock, aapl, facebook stock, googl stock price today, aapl stock, amzn stock price today, alphabet
MSFT	stock msft, msft stock price, msft dividend, msft stock price today, msft stocks, amazon stock, msft ex dividend date, googl stock, djia dow jones industrial average, MSFT

**Table 4:** Google Trends search keywords determined by tweets.

Stock markets/corporations	Keywords
DJIA	dow jones industrial average, industrial average, dow jones, dow jones industrial, dow jones industrial average futures, dow jones industrial average today, spdr dow jones industrial average etf, close of trade, djia dow jones industrial average, u.s. stocks, record high, dow jones industrial average index, wall street, us stocks, jones industrial average, news dow jones industrial average, the dow jones industrial average, new york, stock market, industrial average today, high close, money morning, djia dow jones, dow stocks, dow jones industrial average etf

RUT	russell 2000 index, ishares russell, russell 2000, index etf, short interest, index fund, wave life sciences, union bankshares, small-cap russell, options trade short russell, index trading, unusual options activity, savings institute bank, recent interest, tpi composites, small caps, new study, delta apparel, one chart, index stock, small stocks, index performance, planet payment, jon najarian, trump administration, argos therapeutics, small-cap stocks, index companies, etf trade, small cap russell, index funds, small cap stocks, aqua metals, russell indexes, options new, great dividend stocks, index shares, mbt financial corp, index rise, short term view, index move, index constituents

S&P 500	dow jones, wall street, stock market, bull market, stock market update, e-mini s&p 500, spdr s&p 500, index fund, s&p global, s&p 500, united states, stock market index, market extra, dow jones industrial average, indice s&p 500, us markets, us stocks, trading outlook, technical outlook, u.s. stocks, spy s&p 500, trump rally, dow futures, technical analysis, record highs, record high, market close, bank of America, trading days, futures s&p 500, swing long, stock index, volatility s&p 500, wall st., dish network, market snapshot, dow jones futures, new highs, market correction, el dow jones, straight weeks, top earners, trump's speech, u.s. stock futures, index funds, futures vs, new post, the treasury market, warren buffett, us stock market, new record

VIX	low volatility, volatility index, election volatility, market volatility, implied volatility, cboe volatility index, realized volatility, brexit volatility, low volatility etf, stocks volatility, daily volatility, spy volatility, high volatility, trading video, long volatility, call option, high beta, low volatility index, historical volatility, trading days, vix volatility index, volatility etf, vix index, vix futures, index options, minimum volatility, united states, volatility index etf, stock market, sector etfs, strategy video, volatility etfs, annualized volatility

IXIC	nasdaq composite, nasdaq composite index, composite index, dow jones, record high, wall street, nasdaq index, record close, close report, new York, nasdaq composite stock index, of Nasdaq, stock market update, dow jones industrial average, dow jones industrial, record highs, new record high, ytd dow jones, all-time high, new record, wall st., composite stock index, u.s. stocks, us stocks, new highs

APPL	apple inc., apple iphone, apple watch, apple stock, apple tv, new iphone apple music, apple ios, aapl stock, aapl apple, tim cook, new macbook, apple stock price, apple aapl, apple macbook, iphone sales, apple shares, apple store, apple watch series, apple earnings, apple ceo, new post, apple airpods, apple the iphone, aapl iphone, aapl, next iphone, apple car, aapl earnings, apple tim cook, apple news, apple app store, apple watch sales, aapl shares, new apple watch, apple releases, apple iphone sales, apple new iphone, apple new macbook, next week, iphone 6s, apple ipad, apple new, iphone, apple support, macos sierra, aapl watch, aapl price, touch bar, apple tax, apple event, aapl chart, apple next iphone, apple will, apple patent, apple tv app, apple history, shares of apple, headphone jack, black iphone, apple sales, aapl stock market, apple analyst, new iphones, apple tax ruling, apple stores, apple maps, next apple watch, apple products, steve jobs

GOOGL	alphabet inc., googl google, googl stock, googl shares, googl earnings, google inc., new post, google home, google maps, google googl, google pixel, google stock, google fiber, apple inc., google play, amazon, apple, alphabet stock, tech stocks, alphabet earnings, google earnings, facebook inc., google assistant, alphabet inc. stock, business google, fang stocks, googl search, alphabet googl, google cloud, googl pixel, google parent alphabet, google news, google search, twitter inc., wall street, google stock market

MSFT	microsoft corporation, microsoft windows, microsoft, msft stock, msft, microsoft corp, microsoft surface, microsoft xbox, msft microsoft, microsoft azure, microsoft office, microsoft stock, new xbox, microsoft band, new windows, apple, microsoft edge, microsoft releases, microsoft health, microsoft teams, microsoft cloud, microsoft hololens, microsoft earnings, microsoft store, new Microsoft, new post, tech stocks, windows phone, msft azure, microsoft msft, bill gates, microsoft dynamics, cloud business, microsoft ceo, nokia, surface book, surface, microsoft surface studio, microsoft surface book, windows store, microsoft twitter, Microsoft employees, microsoft shares, new surface, surface studio, msft earnings, anniversary update, microsoft deal, last earnings, satya nadella, stock update, microsoft surface phone, microsoft lumia, microsoft software, cloud services, surfacebook, msft surface, surface phone, business microsoft, microsoft corporation stock, microsoft co, microsoft partners, microsoft cortana, xbox ones, microsoft partner, new features

**Table 5:** Selected keywords obtained from trending searches by using CFS.

Stock markets/corporations	Keywords
DJIA	dow jones industrial average stocks, lowes stock, ftse 100, tenneco stock, dow jones industrial average
RUT	russell 2000 index, etf russell 2000, russell 2000 stock, russell 2000 futures, vanguard russell 2000, s&p 500 index, russell 2000 today
S&P 500	vanguard, s&p 500 returns, s&p 500 price
VIX	volatility, volatility index, s&p 500 vix, vxx
IXIC	stocks, oil nasdaq, oil price, penny stocks Nasdaq
APPL	aapl dividend, amd stock, aapl stock news, amazon stock, aapl option chain, twlo stock, aa stock
GOOGL	amazon stock, amzn stock price today, alphabet
MSFT	amazon stock, msft ex dividend date, dow jones industrial average

**Table 6:** Selected keywords obtained from tweets by using CFS.

Stock markets/corporations	Keywords
DJIA	dow jones industrial average futures, dowjones industrial average, jones industrial average, dow stocks
RUT	russell 2000, short interest, index trading, small caps, small stocks, trump administration
S&P 500	bull market, index fund, dow jones industrial average, technical outlook, record highs, bank of america, market correction, index funds, new post, warren buffett
VIX	volatility index, market volatility, brexit volatility, united states, volatility etfs
IXIC	record close, dow jones industrial average, record highs, all-time high, new record, composite stock index
APPL	apple inc., apple stock, apple watch series, apple earnings, apple app store, iphone 6s, apple support, touch bar, apple tax, aapl chart, apple history
GOOGL	googl earnings, google inc., google pixel, amazon, apple, alphabet earnings, business google, googl search, alphabet googl, google news, google search
MSFT	microsoft, microsoft office, new windows, microsoft teams, microsoft store, tech stocks, msft azure, bill gates, nokia, surface book, windows store, microsoft twitter, last earnings, surface phone, business microsoft

**Table 7:** Selected keywords and historical data attributes obtained from hybrid data I by using CFS.

Stock markets/corporations	Keywords
DJIA	dow jones, closing values, trading volume
RUT	russell 2000, closing values
S&P 500	S&P 500 index, closing values
VIX	minimum values, closing values
IXIC	maximum values, minimum values, closing values
APPL	apple stock, closing values
GOOGL	minimum values, closing values, trading volume
MSFT	microsoft stock, opening values, closing values

**Table 8 tab8:** Selected keywords and historical data attributes obtained from hybrid data II by using CFS.

Stock markets/corporations	Keywords
DJIA	spdr dow jones industrial average etf, us dollar to mexican peso exchange rate, tenneco stock, closing values, trading volume
RUT	etf russell 2000, vanguard russell 2000, russell 2000 stock price, russell 2000 today, closing values
S&P 500	s&p 500 index fund, spdr, s&p 500 companies, closing values
VIX	vix chart, volatility s&p 500, minimum values, closing values
IXIC	penny stocks nasdaq, maximum values, minimum values, closing values
APPL	amd stock, aapl dividend date, amazon stock, twlo stock, aa stock, closing values
GOOGL	minimum values, closing values, trading volume
MSFT	msft ex dividend date, googl stock, dow jones industrial average, opening values, closing values

**Table 9:** Selected keywords and historical data attributes obtained from hybrid data III by using CFS.

Stock markets/corporations	Keywords
DJIA	u.s. stocks, record high, new york, closing values, trading volume
RUT	russell 2000 index, small-cap Russell, index trading, tpi composites, trump administration, options new, closing values
S&P 500	bull market, index fund, technical outlook, record highs, bank of america, closing values
VIX	long volatility, minimum volatility, sector etfs, minimum values, closing values
IXIC	composite index, stock market update, all-time high, new record, closing values
APPL	apple inc., apple earnings, apple app store, iphone 6s, touch bar, apple history, closing values
GOOGL	amazon, alphabet earnings, business google, minimum values, closing values, trading volume
MSFT	microsoft, microsoft teams, new pos, tmsft azure, windows store, last earnings, surface phone, opening values, closing values

**Table 10:** Values of forecasting indices and LSSVR parameters of DJIA.

Data types	*ϒ*	*σ*	MAPE	MAE
GTU	286.7519	1.0311	5.7590	1185.752
Hybrid data I	11.8648	254.2168	0.9355	193.511
Hybrid data I with CFS	31.6798	179.0302	0.8123	167.9605
GTTS	299.8416	148.7305	8.6149	1774.018
GTTS with CFS	299.8510	121.4957	6.3921	1317.742
Hybrid data II	69.1964	216.8693	1.5655	323.6668
Hybrid data II with CFS	263.8273	299.9348	0.7848	161.9244
GTT	299.8481	188.0274	4.4101	912.5717
GTT with CFS	156.0704	213.6443	4.1227	851.3273
Hybrid data III	239.8938	299.8602	0.8263	170.6021
Hybrid data III with CFS	41.3638	162.1620	0.7903	163.2619
Historical data	8.9820	212.0598	0.9360	193.6717

**Table 11 tab11:** Values of forecasting indices and LSSVR parameters of RUT.

Data types	*ϒ*	*σ*	MAPE	MAE
GTU	28.7932	1.0322	4.1351	57.26787
Hybrid data I	47.1233	280.9206	0.6887	9.558448
Hybrid data I with CFS	187.6684	299.9894	0.6446	8.944846
GTTS	299.6459	58.3971	3.8909	54.00228
GTTS with CFS	288.4127	1.6575	4.7660	66.03728
Hybrid data II	299.8630	298.7397	0.8047	11.17783
Hybrid data II with CFS	242.8707	258.5781	0.5860	8.132567
GTT	142.0803	299.9982	6.0535	83.95828
GTT with CFS	299.2949	36.2646	4.5243	62.78645
Hybrid data III	275.9271	299.9795	4.4557	61.93947
Hybrid data III with CFS	290.5348	299.9864	0.6833	9.474567
Historical data	15.9065	212.8006	0.6543	9.062642

**Table 12 tab12:** Values of forecasting indices and LSSVR parameters of S&P 500.

Data types	*ϒ*	*σ*	MAPE	MAE
GTU	286.0031	1.3630	5.2607	123.5923
Hybrid data I	296.4163	21.8955	2.4812	58.47516
Hybrid data I with CFS	299.9114	299.7517	0.3227	7.563877
GTTS	299.9900	178.2046	2.5083	58.97946
GTTS with CFS	299.1337	53.7133	3.0600	71.94199
Hybrid data II	81.3331	299.3654	0.7000	16.47316
Hybrid data II with CFS	299.8863	42.0948	0.4111	9.662183
GTT	295.725783	299.853066	3.5048	82.56633
GTT with CFS	299.736734	28.149033	4.3884	103.0099
Hybrid data III	294.392728	299.987257	2.4173	57.06461
Hybrid data III with CFS	299.833343	217.828272	1.0020	23.61675
Historical data	276.3041	117.9110	0.7883	18.59049

**Table 13 tab13:** Values of forecasting indices and LSSVR parameters of VIX.

Data types	*ϒ*	*σ*	MAPE	MAE
GTU	13.3603	1.1941	14.1340	1.6245
Hybrid data I	158.7245	299.7233	3.8714	0.4455
Hybrid data I with CFS	299.9185	164.9604	3.6115	0.4164
GTTS	2.4697	36.8100	16.2766	1.8678
GTTS with CFS	299.9363	163.1365	15.3256	1.7562
Hybrid data II	94.8736	299.7342	4.3329	0.5021
Hybrid data II with CFS	164.3688	299.2222	3.9557	0.4553
GTT	17.6774	299.8496	17.4122	1.9839
GTT with CFS	47.4322	7.8556	17.9481	2.0552
Hybrid data III	25.4548	299.5653	4.3524	0.5017
Hybrid data III with CFS	299.4207	204.1631	3.4112	0.3945
Historical data	299.8795	265.0368	3.9465	0.4540

**Table 14 tab14:** Values of forecasting indices and LSSVR parameters of IXIC.

Data types	*ϒ*	*σ*	MAPE	MAE
GTU	299.3550	2.5105	6.6582	386.1416
Hybrid data I	272.0069	299.9858	0.3309	19.1456
Hybrid data I with CFS	298.6805	299.9605	0.3755	21.7364
GTTS	299.6967	40.4296	4.3866	254.6377
GTTS with CFS	299.9400	14.9974	1.7051	99.0539
Hybrid data II	215.4590	299.9670	0.6410	37.0282
Hybrid data II with CFS	299.8119	299.9796	0.3200	18.5165
GTT	278.2689	171.0348	6.3860	370.6502
GTT with CFS	297.1919	12.0165	7.4833	433.7651
Hybrid data III	242.2756	299.9197	0.6113	35.2682
Hybrid data III with CFS	299.8552	113.7700	0.7048	40.7125
Historical data	297.5621	299.8541	0.3524	20.3881

**Table 15 tab15:** Values of forecasting indices and LSSVR parameters of the Apple corporation.

Data types	*ϒ*	*σ*	MAPE	MAE
GTU	299.654	2.026	16.549	22.304
Hybrid data I	299.868	58.567	6.432	8.744
Hybrid data I with CFS	296.624	3.189	14.037	19.054
GTTS	299.393	80.071	15.437	20.895
GTTS with CFS	299.623	284.702	14.015	18.947
Hybrid data II	299.934	299.323	7.197	9.786
Hybrid data II with CFS	299.994	185.826	2.739	3.712
GTT	289.855	4.202	15.845	21.417
GTT with CFS	151.192	8.150	14.463	19.556
Hybrid data III	290.552	299.978	13.824	18.680
Hybrid data III with CFS	297.215	299.939	2.371	3.147
Historical data	298.335	14.179	10.233	13.967

**Table 16 tab16:** Values of forecasting indices and LSSVR parameters of the Alphabet corporation.

Data types	*ϒ*	*σ*	MAPE	MAE
GTU	15.835	56.168	2.739	3.712
Hybrid data I	86.419	299.910	0.440	3.689
Hybrid data I with CFS	113.595	299.957	0.480	4.027
GTTS	2.094	298.483	4.581	38.667
GTTS with CFS	295.316	20.147	4.030	34.086
Hybrid data II	265.706	299.892	1.146	9.585
Hybrid data II with CFS	113.288	299.833	0.480	4.027
GTT	1.001	284.345	4.383	37.005
GTT with CFS	80.220	3.854	4.730	39.973
Hybrid data III	33.772	274.467	2.162	18.136
Hybrid data III with CFS	135.191	299.891	0.463	3.894
Historical data	299.903	17.300	0.442	3.719

**Table 17:** Values of forecasting indices and LSSVR parameters of the Microsoft Corporation.

Data types	*ϒ*	*σ*	MAPE	MAE
GTU	289.734	2.215	7.243	4.670
Hybrid data I	299.981	279.951	0.560	0.361
Hybrid data I with CFS	295.796	299.845	0.577	0.372
GTTS	298.544	3.452	6.011	3.870
GTTS with CFS	299.382	1.059	6.391	4.120
Hybrid data II	296.317	299.957	1.254	0.809
Hybrid data II with CFS	296.490	16.132	1.425	0.919
GTT	291.903	18.957	7.274	4.685
GTT with CFS	43.700	10.620	6.119	3.941
Hybrid data III	294.953	299.952	6.764	4.355
Hybrid data III with CFS	296.273	299.994	0.912	0.589
Historical data	113.445	2.280	0.727	0.469

## Data Availability

The data used to support the findings of this study are included within the article by website linkages.
